# Supplementary material for: Conditions to Control Furan Ring Opening during Furfuryl Alcohol Polymerization
Source: Molecules. 2022 May 17;27(10):3212. doi: 10.3390/molecules27103212 (PMC9145036; doi:10.3390/molecules27103212)
Supplement: Supplementary file 1 [file molecules-27-03212-s001.zip › molecules-1711881-supplementary.pdf]

Supplementary materials

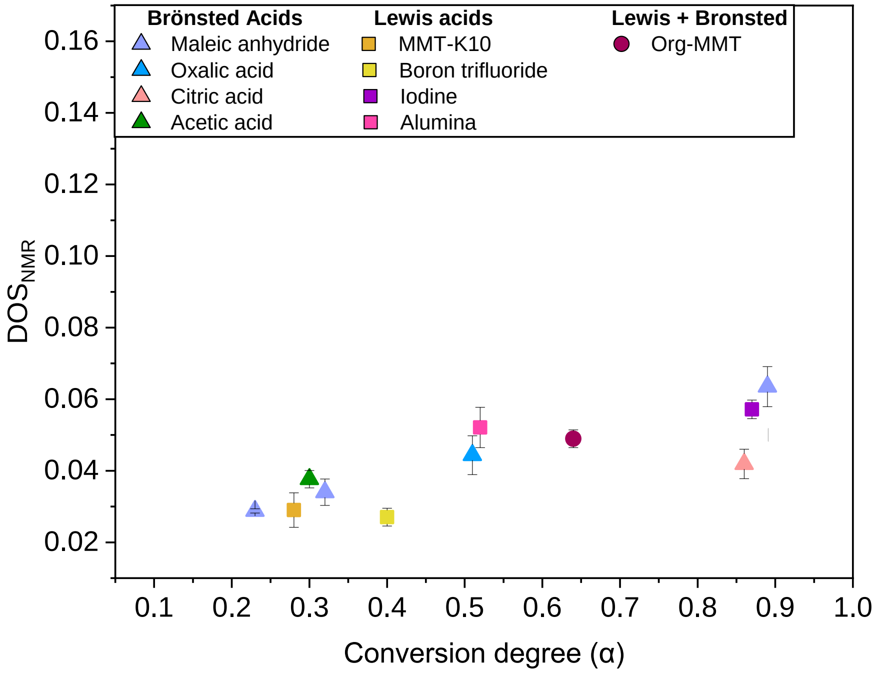

Figure S1. Comparison of the degree of open structures obtained from ^{19}F NMR method synthesized with different initiators in function of the conversion degree.

Table S1. Index of the results of the different initiators used without water.

Initiator	Acid type	DOS_{NMR} / DOS_{Titri}	DOS_{Titri}	DOS_{NMR}	α	FTIR C=O area
Citric acid	Bronsted	0.68	0.061	0.042	0.86	17.9
Oxalic acid	Bronsted	0.93	0.047	0.044	0.51	8.50
Nitric acid	Bronsted	0.63	0.108	0.068	0.4	8.07
Acetic acid	Bronsted	0.37	0.078	0.029	0.3	9.5
Maleic anhydride	Bronsted	0.70	0.092	0.064	0.89	21.05
Maleic anhydride	Bronsted	0.54	0.054	0.029	0.23	5
Maleic anhydride	Bronsted	0.60	0.057	0.034	0.32	6.1
Org-MMT 2 wt%	Lewis / Bronsted	0.67	0.030	0.020	0.53	2.33
Org-MMT 2 wt%	Lewis / Bronsted	0.52	0.095	0.049	0.64	11.53
Org-MMT 1 wt%	Lewis / Bronsted	0.53	0.040	0.021	0.14	3.85
MMT K10 2 wt%	Lewis	0.39	0.075	0.029	0.28	7.39
Boron trifluoride	Lewis	0.48	0.056	0.027	0.4	5.86
Alumina	Lewis	0.55	0.094	0.052	0.51	30.39
Iodine	Lewis	0.78	0.073	0.057	0.87	19.13

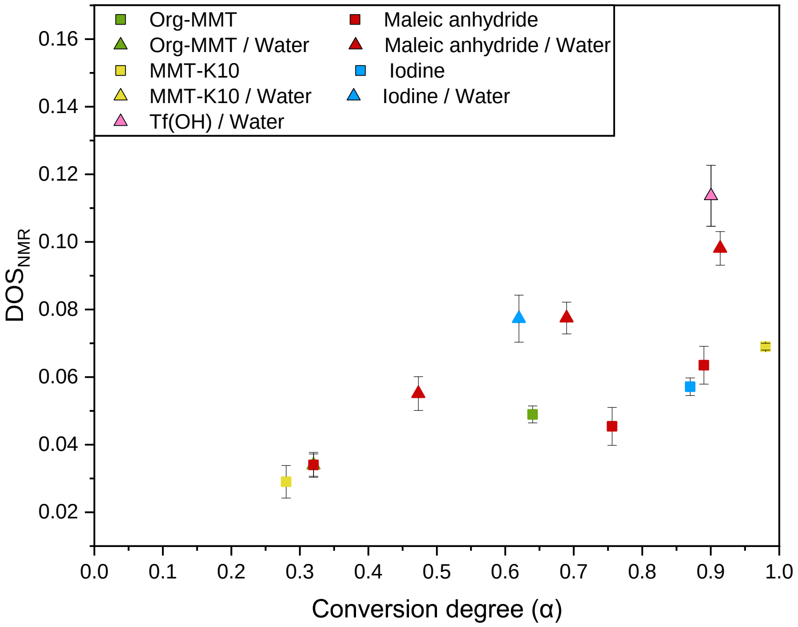

Figure S2. Comparison of the degree of open structures obtained ^{19}F NMR method and synthesized with different initiators with and without additional water (50 % w/w) in function of the conversion degree.

Table S2. Index of the results of the different initiators used without water.

**Conditions**	**Acid type**	**DOS$_{NMR}$ / DOS$_{Titri}$**	**DOS$_{Titri}$**	**DOS$_{NMR}$**	**α**	**IR area**
Maleic anhydride neat	Brönsted	0.60	0.057	0.034	0.32	6.1
Maleic anhydride neat	Brönsted	0.54	0.083	0.045	0.76	13.48
Maleic anhydride neat	Brönsted	0.70	0.092	0.064	0.89	21.05
Maleic anhydride with water	Brönsted	0.71	0.077	0.055	0.47	8
Maleic anhydride with water	Brönsted	0.73	0.107	0.078	0.69	12.15
Maleic anhydride with water	Brönsted	0.71	0.139	0.098	0.91	25.96
Org-MMT (2 wt %) neat	Lewis / Brönsted	0.52	0.095	0.049	0.64	11.53
Org-MMT (2 wt %) with water	Lewis / Brönsted	0.49	0.069	0.034	0.32	2.33
Iodine neat	Lewis	0.91	0.073	0.057	0.87	19.13
Iodine with water	Lewis	0.78	0.099	0.077	0.62	16.88
MMT-K10 neat	Lewis	0.41	0.071	0.029	0.28	7.39
MMT-K10 with water	Lewis	0.56	0.123	0.069	0.98	12.53

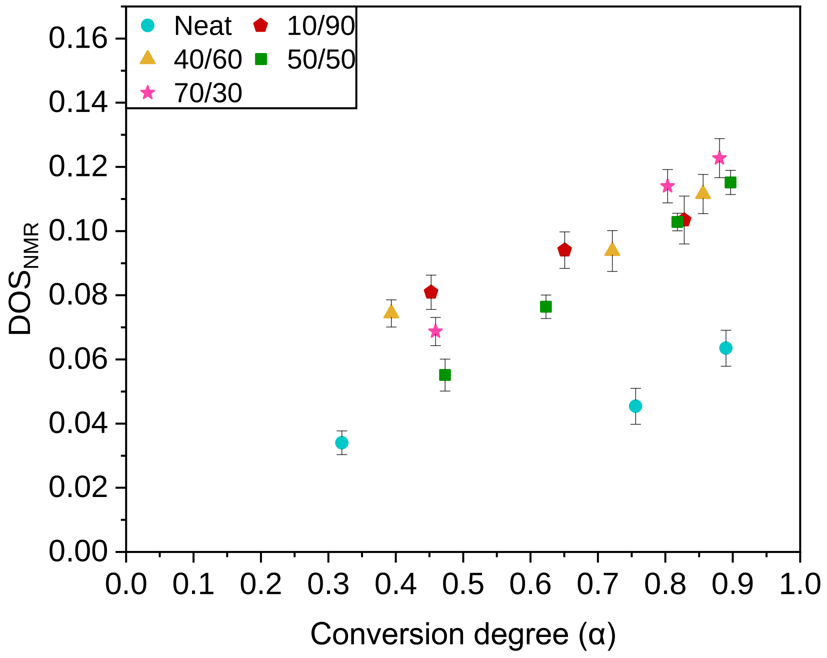

Figure S3. Comparison of the degree of open structures obtained with ^{19}F NMR method and synthesized with different FA/Additional water ratios with in function of the conversion degree.

Table S3. Index of the results of the different FA/additional water ratios used with maleic anhydride.

FA/ Water ratio (w/w)	DOS_{NMR} / DOS_{Titri}	DOS_{Titri}	DOS_{NMR}	α	IR area
Neat	0.60	0.057	0.034	0.32	6.1
Neat	0.54	0.083	0.045	0.76	13.48
Neat	0.70	0.092	0.064	0.89	21.05
70/30	0.75	0.099	0.074	0.39	11.24
70/30	0.75	0.126	0.094	0.72	15.6
70/30	0.72	0.156	0.112	0.86	21.89
50/50	0.71	0.077	0.055	0.47	8
50/50	0.73	0.107	0.078	0.69	12.15
50/50	0.71	0.139	0.098	0.91	25.96
40/60	0.74	0.093	0.069	0.46	10.46
40/60	0.93	0.123	0.114	0.80	16.94
40/60	0.82	0.150	0.123	0.88	20.26
10/90	0.702	0.115	0.081	0.45	12.97
10/90	0.710	0.132	0.094	0.65	19.9
10/90	0.665	0.155	0.103	0.83	32
